# Editorial: Microbial Biofilms in Chronic and Recurrent Infections

**DOI:** 10.3389/fmicb.2021.803324

**Published:** 2021-11-30

**Authors:** Fiorentina Ascenzioni, Axel Cloeckaert, Enea Gino Di Domenico, Catherine Dunyach-Remy, María Guembe

**Affiliations:** ^1^Department of Biology and Biotechnology “Charles Darwin”, Sapienza University of Rome, Rome, Italy; ^2^INRAE, Université de Tours, UMR, ISP, Nouzilly, France; ^3^Microbiology and Virology, IRCCS San Gallicano Institute, Rome, Italy; ^4^VBIC, INSERM U1047, Université de Montpellier, Service de Microbiologie et Hygiène Hospitalière, CHU Nîmes, Nîmes, France; ^5^Instituto de Investigación Sanitaria Gregorio Marañón, Madrid, Spain; ^6^Department of Clinical Microbiology and Infectious Diseases, Hospital General Universitario Gregorio Marañón, Madrid, Spain

**Keywords:** biofilm, infection, inflammation, resistance, antibiotic

Biofilm plays a significant role in the pathogenesis of most chronic infections in humans, either tissue-specific or involving medical implants (Lebeaux et al., 2014). Biofilm-associated infections exhibit high resistance to host defenses, often contributing to an excessive or inappropriate inflammatory response leading to further tissue damage and spreading of the infection (Jensen et al., 2010). On the other hand, biofilms are highly tolerant to antimicrobial therapy (Römling and Balsalobre, 2012; Di Domenico et al., 2019). Biofilms can tolerate up to 100–1,000 times higher minimal inhibitory concentration (MIC) than the same bacterial cells in planktonic growth (Macià et al., 2014). Unfortunately, the effective antibiotic MIC *in vivo* for biofilm eradication may be impossible to reach due to the drugs' toxicity and side effects, including limitations imposed by renal and/or hepatic functions (Ciofu et al., 2015). However, *in vitro* experiments showed that an aggressive antibiotic treatment can effectively eradicate biofilm during the initial stage of colonization (Lebeaux et al., 2014; Ciofu et al., 2015). Despite their importance, the early recognition of biofilm-associated infections still represents an unmet need in clinical microbiology. Therefore, the development of novel diagnostic and therapeutic strategies is urgently needed to manage biofilm-associated infections effectively.

We thank all the authors who contributed with their relevant works to this Research Topic by analyzing different aspects of the pathogenesis and therapeutic management of chronic biofilm-related infections.

In their review paper, Huang et al., examined the most recent metagenomic and metatranscriptomic evidence to study the links between microbial dysbiosis and periodontitis. It has been well-established that polymicrobial biofilms play an essential role in the initiation and progression of periodontitis. In particular, an imbalanced microbial community alters the host response, leading to inappropriate inflammation, further damaging periodontal tissues. In turn, the inflammatory response affects the oral microbiome and modulates the expression of bacterial virulence factors. The authors concluded that assessing the correlation between the dynamic changes in the functional behaviors of the periodontal microbiome and the progression and outcome of periodontitis might provide valuable information for developing effective prevention and treatment strategies.

Dental caries is another typical biofilm-related disease of the oral cavity, and the acidification of biofilm pH is central in diseases development. Zhang et al. showed that pH-responsive core-shell nano micelle loaded with bedaquiline, can potently inhibit the growth of *Streptococcus mutans* biofilm in an acidic environment (pH 5) without cytotoxic effect on the periodontal cells. Therefore, this pH-responsive micelle showed great potential in preventing dental caries.

Biofilm formation on implant surfaces is considered the main risk factor for inflammatory tissues processes and implant failure. However, in many cases, traditional cultures have proven poor sensitivity in pathogen isolation. In their paper, Oliva, Miele et al. provided a critical overview on the principal culture-based methods, sonication technique molecular testing used in the clinical practice for the most effective identification of the causative agents of implant-associated infections.

Biofilm-growing *Staphylococcus aureus* is a prominent cause of implant-associated infections. The study conducted by Yu et al. showed that *S. aureus* nucleases nuc1 and nuc2 are complementary genes involved in biofilm formation in implant-associated infections, with nuc2 contributing less to virulence and immune evasion.

*S. aureus* is also the most feared pathogen in another clinically relevant infection like a prosthetic joint infection. The study presented by Lamret et al. has hypothesized that the periprosthetic bone environment is stressful for *S. aureus*, influencing biofilm development. The authors assessed several parameters to mimic bone environment, such as lack of oxygen, casamino acids and glucose starvation, and high magnesium concentration. In the classic *in vitro* model, the *S. aureus* strains formed various biofilm structures. However, in the bone-like environment, these strains shared common responses, including the increase in biofilm biomass and number of adherent cells proportion with a similar production of extracellular DNA and engagement of the SOS response. By mimicking the highly challenging bone-like conditions, this model may provide the base for future anti-biofilm strategies.

In an *in vitro* model simulating a prosthetic joint infection, Scheper et al. evaluated the effects of antimicrobial peptides on Methicillin-resistant *S. aureus* (MRSA) persisters exposed to antibiotics. The combined activity of rifampicin and ciprofloxacin alone did not eliminate persister cells within mature biofilms grown on polystyrene plates, titanium/aluminum/niobium disks, and prosthetic joint liners. However, SAAP-148, a synthetic peptide based on LL-37, eradicated persisters within mature biofilms on different abiotic surfaces. Based on these data, SAAP-148 resulted in a promising candidate for further development as an agent for the treatment of biofilm-associated prosthetic joint infection.

MRSA isolates are of particular concern in patients with cystic fibrosis. Biofilm formation is a common characteristic of pathogenic MRSA strains representing a critical barrier to eradication treatment and host defenses. In their study, Boudet et al. investigated, by the Antibiofilmogram®, the ability of MRSA isolates from patients with cystic fibrosis to form a biofilm with and without antibiotics. They highlighted that ceftaroline and ceftobiprole were remarkably active in hampering the biofilm formation of certain strains adapted to the lung environment of patients with cystic fibrosis. In addition, the authors concluded that the Antibiofilmogram® could be a promising tool for guiding the choice of the most effective drugs against biofilm-growing MRSA in CF airways.

The lipoglycopeptide dalbavancin emerged as another promising agent for treating biofilm-related infections. In the review article presented by Oliva, Stefani et al. the authors outlined the mechanisms regulating biofilm development in *Staphylococcus* and *Enterococcus* species and the clinical impact of biofilm-related infections. Thus, the authors conclude that the use of dalbavancin may represent a valuable option for the treatment of skin and soft tissues infections and other invasive Gram-positive infections.

Biofilm plays a significant role in the progression and chronicity of diabetic foot ulcers. In their review article, Pouget et al. discussed current knowledge and the contribution of biofilms on diabetic foot ulcers. In particular, the authors focused on preventive strategies to hinder the establishment of microbial biofilms and wound chronicity to support or eventually replace the current approach for managing diabetic foot ulcers.

Biofilms contribute to the pathogenesis of several otorhinolaryngologic chronic infective disorders, including otitis media, chronic rhinosinusitis, and cholesteatoma. Patients with acquired cholesteatoma generally present with chronic otorrhea and progressive conductive hearing loss. Jiang et al. highlighted by next-generation sequencing (NGS)-based approach that the severity of the patient's pathological condition worsens the more complex the types of microbes' infections. The results obtained from this study revealed that the most commonly detected microbial genus was *Aspergillus*, particularly in patients with severe bone erosion. In summary, NGS help to identify pathogens of cholesteatoma patients and *Aspergillus* infections in acquired cholesteatoma.

Finally, we would like to thank the people that supported this Research Topic and the reviewers for their time and comments that helped to improve the manuscripts.

## Author Contributions

All authors have contributed substantially to the manuscript, providing a direct, and intellectual contribution to the work. All authors approved the final version of the manuscript.

## Conflict of Interest

The authors declare that the research was conducted in the absence of any commercial or financial relationships that could be construed as a potential conflict of interest.

## Publisher's Note

All claims expressed in this article are solely those of the authors and do not necessarily represent those of their affiliated organizations, or those of the publisher, the editors and the reviewers. Any product that may be evaluated in this article, or claim that may be made by its manufacturer, is not guaranteed or endorsed by the publisher.
